# In Vitro Evaluation of Time-Dependent Linear Dimensional Changes in Four Interocclusal Recording Materials

**DOI:** 10.7759/cureus.89819

**Published:** 2025-08-11

**Authors:** Vandana Rai, Mohammed Aleemuddin, Kanimozhi Kulasekaran, Sushant A Pai, Ananya Singh

**Affiliations:** 1 Prosthodontics, Crown and Bridge, Sree Balaji Dental College and Hospital, Chennai, IND; 2 Conservative Dentistry and Endodontics, Yenepoya Dental College, Mangalore, IND; 3 Dentistry and Epidemiology, Private Practice, Chennai, IND; 4 Prosthodontics, Sri Rajiv Gandhi College of Dental Science and Hospital, Bengaluru, IND; 5 Prosthodontics, Rajendra Institute of Medical Sciences, Ranchi, IND

**Keywords:** bite registration, dimethacrylate, elastomers, impression material, interocclusal records, linear dimensional change, oral and maxillofacial prosthesis, oral rehabilitation, poly ether, polyvinylsiloxane

## Abstract

Aim: This study aimed to evaluate and compare the linear dimensional and weight changes of four types of interocclusal recording materials, polyether (Ramitec), two polyvinylsiloxane variants (O-Bite and Jet Bite), and dimethacrylate (LuxaBite), at 1-hour, 24-hour, and 1-week intervals to assess their effectiveness in maintaining accurate occlusal records.

Materials and methods: 20 samples of each interocclusal recording material were prepared using a stainless-steel die with a test block and ring mold. Each sample, measuring 30 mm in diameter and 2 mm in thickness, was subjected to a 500-g weight for five minutes. Dimensional changes were measured using a stereomicroscope, and weight changes were assessed with an electronic scale at the specified intervals of 1 hour, 24 hours, and 1 week. Statistical analyses such as one-way analysis of variance (ANOVA) and repeated measures ANOVA, were used to compare the data using SPSS software version 26.

Results: Among the materials evaluated, polyvinylsiloxane material (Jet Bite) demonstrated the highest accuracy and dimensional stability with minimal changes over time (0.49% at 1 hour, 0.52% at 24 hours, and 0.50% at 1 week). The second polyvinylsiloxane (O-Bite) exhibited slightly higher dimensional changes (1.13% at 1 hour, 1.51% at 24 hours, and 1.50% at 1 week). The polyether material (Ramitec) showed significant initial variation (3% at 1 hour) but minimal changes thereafter. The dimethacrylate material (LuxaBite) expanded at 1 hour and then contracted, correlating with weight loss due to polymerization.

Conclusion: Among the materials tested, polyvinylsiloxane demonstrated the highest dimensional accuracy and stability. Accuracy was markedly improved when casts were mounted shortly after recording, highlighting the importance of minimizing storage time. Additionally, all materials exhibited a significant correlation between weight changes and dimensional alterations, suggesting that shrinkage or expansion can impact clinical reliability. Therefore, selecting appropriate recording materials and ensuring timely mounting of the cast are crucial for achieving optimal prosthodontic results.

## Introduction

Accurate positioning of dental casts is vital for diagnosis, treatment planning, and prosthetic rehabilitation. While manual stabilization can sometimes position the casts in maximum intercuspation, interocclusal recording materials are often necessary to transfer the maxillomandibular relationship accurately to the articulator [1]. Interocclusal records aid clinicians in fabricating prostheses with minimal adjustment saving valuable chair side time and reducing the need for clinical and technical revisions.

Since the inception of casting models for dentures in the 18th century, interocclusal recording materials have evolved, including impression plaster, waxes, zinc oxide eugenol, and acrylic resins [2]. More recently, elastomeric materials have been developed for their improved dimensional stability and handling. Ideally, these materials must be easy to use, tissue-friendly, dimensionally stable, and able to accurately reproduce occlusal surfaces [3].

Selecting an appropriate interocclusal recording material is crucial for maintaining dimensional stability, especially in clinical settings where casts are sent to external dental laboratories. During storage and transportation, these materials must retain their accuracy to ensure reliable and precise outcomes, which are fundamental for the fabrication of well-fitting prostheses. However, dimensional alterations may occur due to factors such as polymerization shrinkage, moisture absorption, or the release of volatile byproducts. Therefore, it is essential to use materials that exhibit minimal changes in dimension and weight over time.

This study aimed to assess and compare the time-dependent dimensional and weight changes of four interocclusal recording materials (polyether, two polyvinylsiloxanes, and dimethacrylate) with the purpose of evaluating their stability, accuracy, and clinical reliability. The objective further emphasizes understanding the influence of polymerization characteristics and moisture interaction on material behavior, to support evidence-based selection of suitable materials for accurate occlusal record transfer, particularly in cases where immediate cast articulation is not feasible.

## Materials and methods

This in vitro study was conducted in the Department of Prosthodontics and Crown & Bridge, Sri Rajiv Gandhi College of Dental Sciences and Hospital, Bengaluru, India.

Sample size calculation

The sample size for this in vitro study was based on previous research by Michalakis et al. and Gurav et al., who assessed the dimensional stability of interocclusal materials using 10 to 20 specimens per group [1,2]. Following this approach and to ensure statistically valid comparisons, 20 samples were included per group, resulting in a total of 80 specimens. This number was sufficient to detect meaningful differences with a statistical power of 80% at a 0.05 significance level using one-way analysis of variance (ANOVA).

Materials used

This study evaluated four widely used interocclusal recording materials: a polyether material (Ramitec), two polyvinylsiloxane bite registration pastes, and a dimethacrylate material (LuxaBite) (Table 1).

**Table 1:** Commercially available interocclusal recording materials used in the study Table credits: Kanimozhi Kulasekaran

Type of Material	Product Name	Group	Manufacturer (Headquarters)
Polyether	Ramitec	A	3M ESPE, Seefeld, Germany
Polyvinylsiloxane	O-Bite	B	DMG Chemisch-Pharmazeutische Fabrik GmbH, Hamburg, Germany
Polyvinylsiloxane	Jet Bite	C	Coltène/Whaledent AG, Altstätten, Switzerland
Dimethacrylate	LuxaBite	D	DMG Chemisch-Pharmazeutische Fabrik GmbH, Hamburg, Germany

Using a standardized test setup comprising a ring mold and a stainless-steel die with a test block, specimens of four different interocclusal recording materials were prepared. 20 samples from each material group were fabricated. Each sample measured 30 mm in diameter and 2 mm in thickness, and was subjected to a constant load of 500 g for five minutes to facilitate uniform setting. After removal from the die, the linear dimensional change was assessed by measuring the distance between two reference points (A and B) on the test block at three time intervals: 1 hour, 24 hours, and 1 week. These measurements were obtained using a zoom stereo microscope (MZS0745, Metazoom, India) with a magnification range of 3.5X to 45X. In parallel, an electronic precision scale was used to record the weight of each specimen at the same time intervals to evaluate weight changes over time.Statistical analyses were performed using SPSS software version 26 (IBM Corp., USA). Prior to applying parametric tests, the normality of the data was evaluated using the Shapiro-Wilk test. As all datasets were normally distributed (p>0.05) One-way ANOVA and repeated measures ANOVA were used to assess mean differences between groups and across time points. A p-value less than 0.05 was considered statistically significant.

Inclusion criteria

The samples with uniform 2 mm thickness, consistent color, and free from visible lines or voids were included.

Exclusion criteria

Those samples with inconsistencies or defects were excluded.

Fabrication of the die

**Figure 1:**
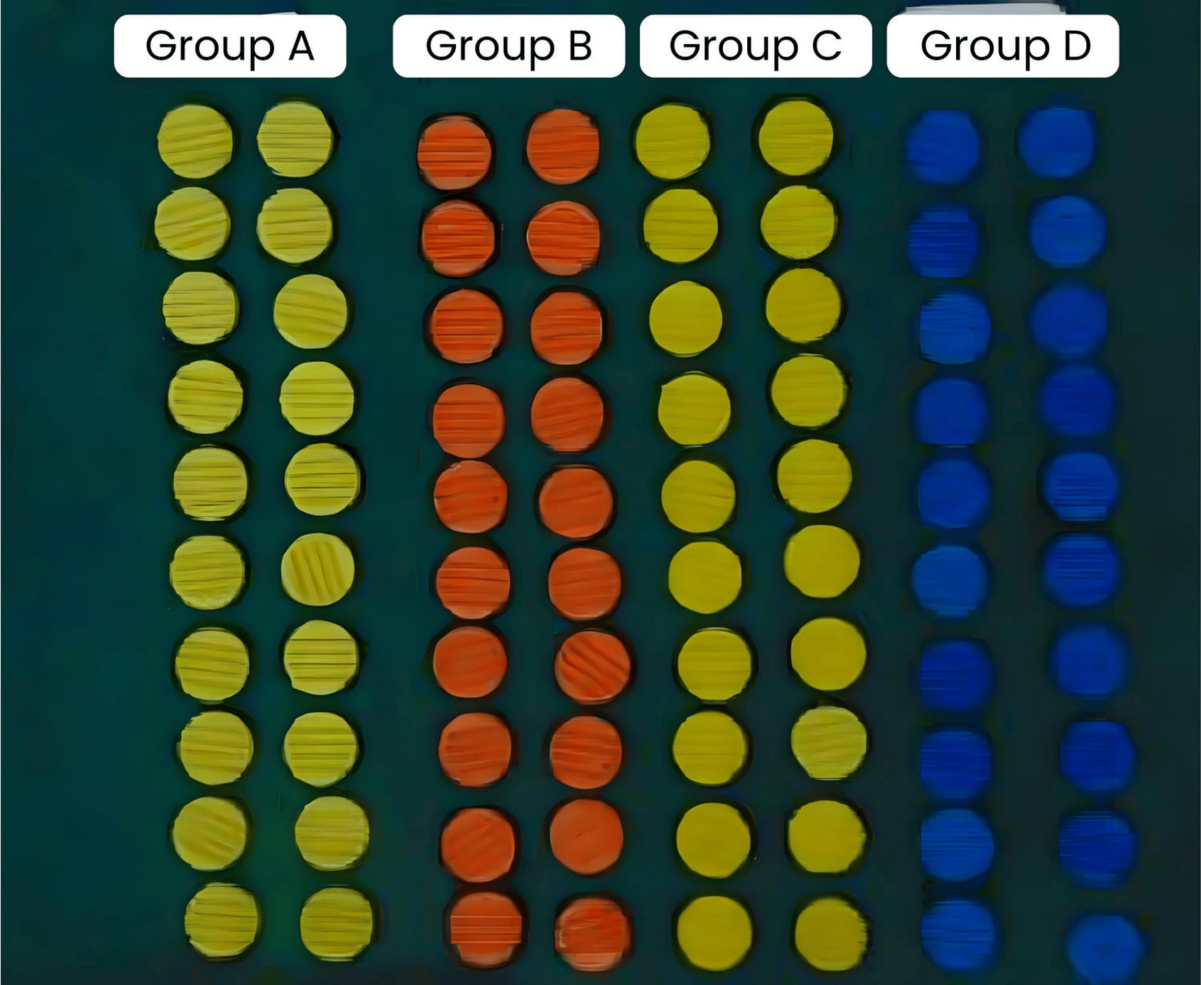
Total number of samples (80 samples) Figure credits: Vandana Rai

**Figure 2:**
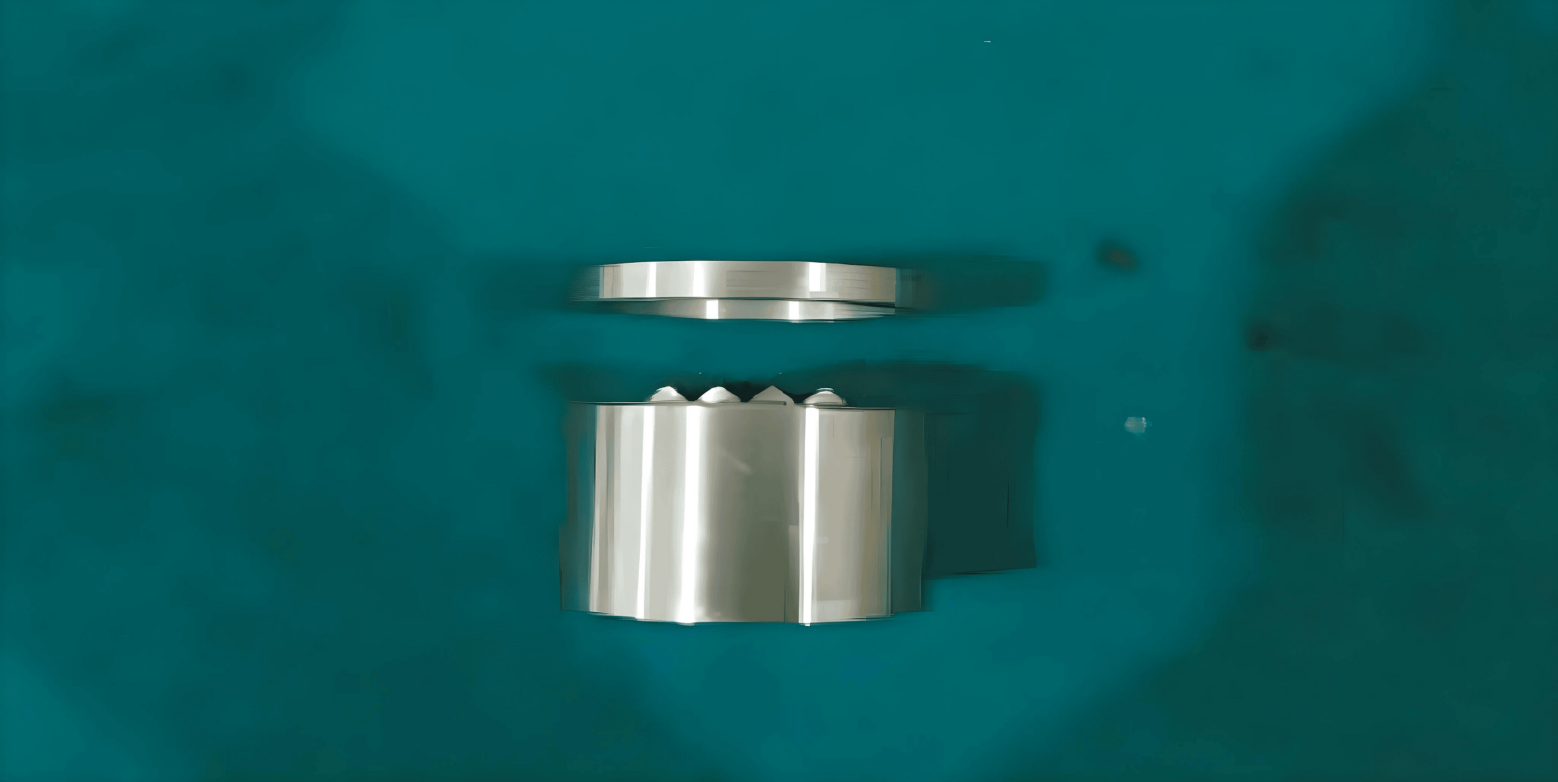
Stainless-steel die, consisting of a test block and a ring block Figure credits: Vandana Rai

**Figure 3:**
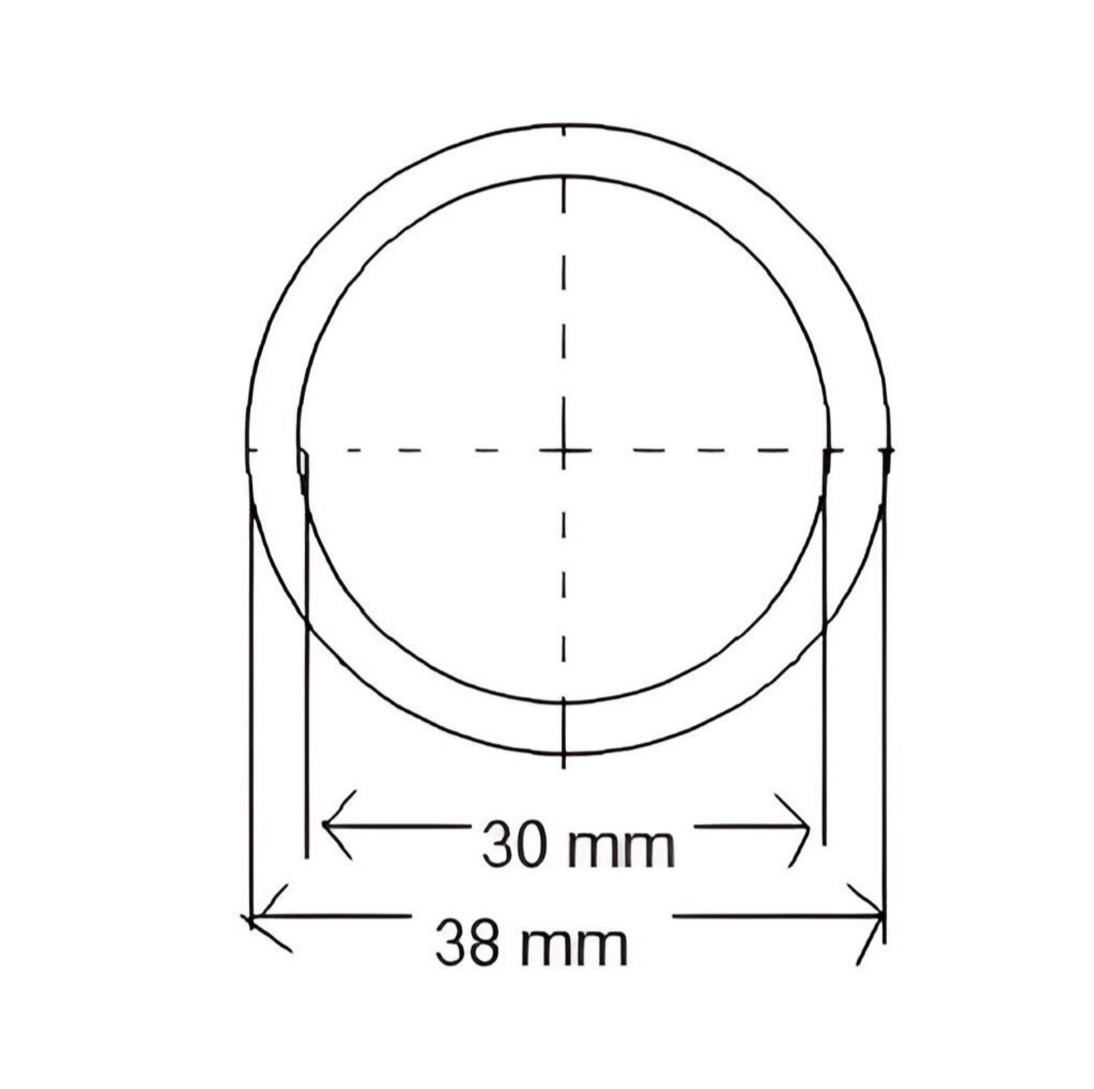
Illustration for the external and internal dimensions of the stainless steel mold Figure credits: Vandana Rai

**Figure 4:**
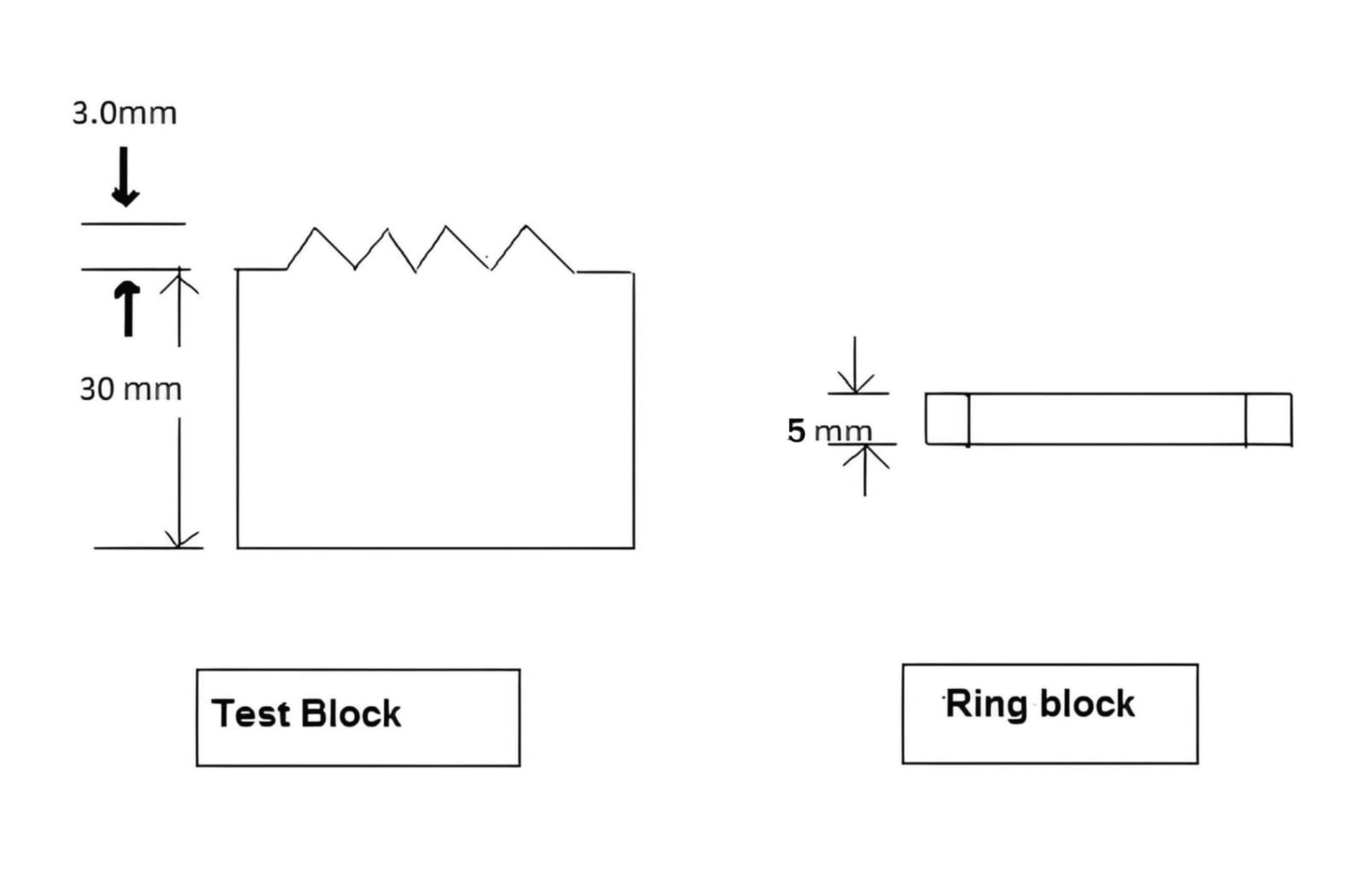
Diagram illustrating the test block and ring block Figure credits: Vandana Rai

Each group comprised 20 samples, resulting in a total of 80 samples (Figure 1) were prepared using a stainless-steel die (Figure 2). The inner dimensions of the cylinder-shaped ring mold were 30 mm in diameter and 2 mm in thickness (Figure 3). The die consisted of a test block (Part A) and a ring mold (Part B) (Figure 4). Points A and B in the test block served as reference guides, with the distance between these points being 20.11 mm.

Manipulation of the materials

Polyether (Ramitec)

Equal parts of base and catalyst were hand-mixed for 30 seconds, loaded into a syringe, and applied to the die without air entrapment.

Polyvinylsiloxane Materials (O-Bite, Jet Bite) and Dimethacrylate (LuxaBite)

These materials were dispensed using auto-mixing cartridges with attached mixing tips, ensuring even application onto the die surfaces, taking care to avoid air bubbles for accurate registration.

Preparation of samples

Each material was applied to a stainless steel die and allowed to set for five minutes under a 500-g load on a glass plate covered with a polyethylene sheet, resulting in a total applied force of 5.56 N, including the plate’s weight (67 g). This standardized pressure was used to counteract the initial resistance of the interocclusal materials, which typically ranges from 0.5 N to 13.8 N. After setting, the samples were carefully removed and excess material was trimmed using a Bard Parker blade. Each sample measured 30 mm in diameter and 2 mm in thickness. A total of 80 samples were prepared, 20 from each of the four material groups, and stored at room temperature in non-hygroscopic polyethylene bags between observation intervals.

Measurements and statistical analysis

Equipment Used

**Figure 5:**
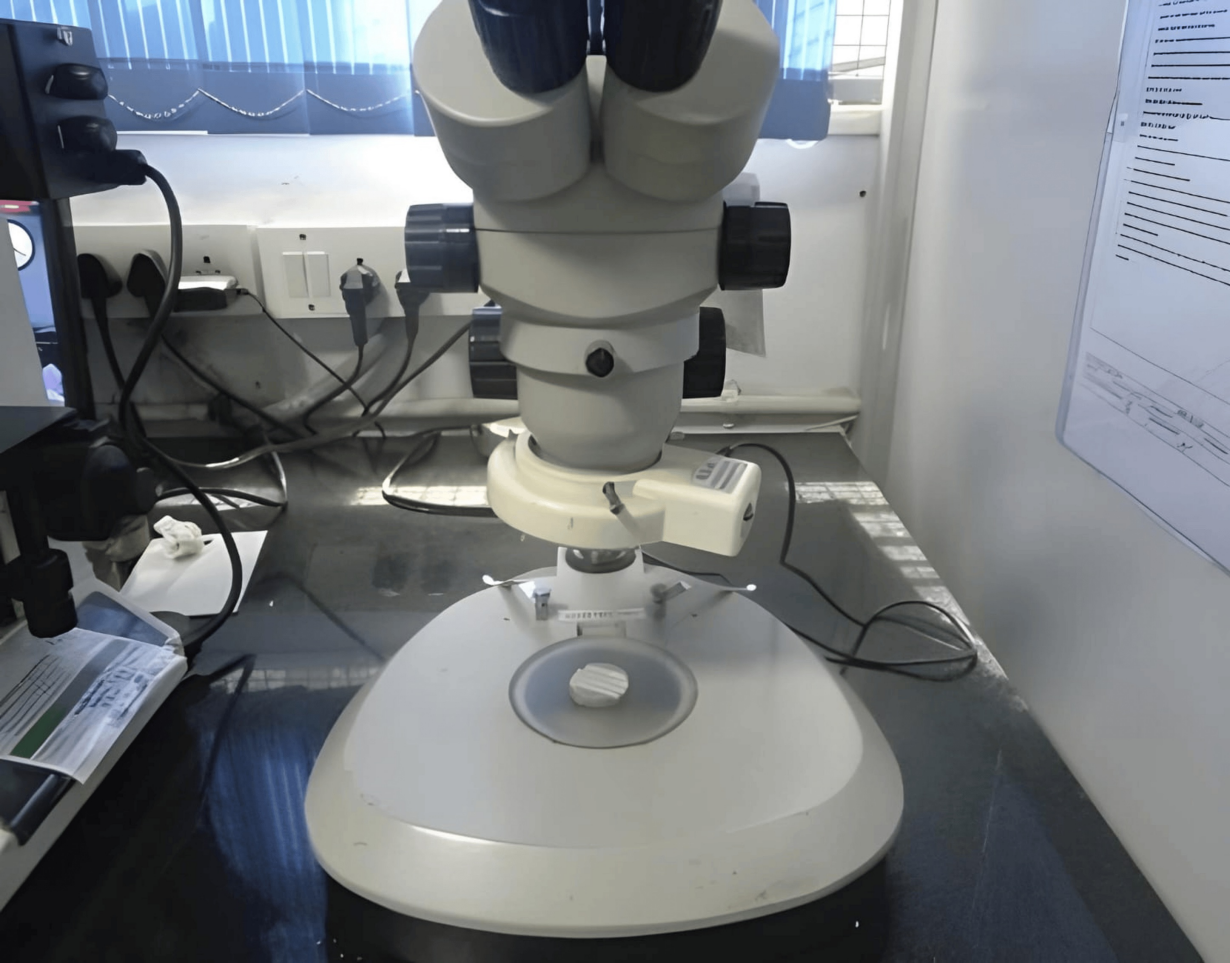
Stereomicroscope Figure credits: Vandana Rai

A zoom stereomicroscope (MZS0745) with a magnification range of 3.5X to 45X was used for both visual inspection and precise linear dimensional measurements. This magnification range is suitable for detailed examination of surface features and microstructural characteristics of the interocclusal recording materials (Figure 5). The stereomicroscope was calibrated using a certified linear glass scale (range: 0-10 mm, least count: 0.1 mm). All assessments were performed by a single trained examiner to ensure measurement consistency and reliability.

Linear Dimensional Changes among the Materials During the Intervals

**Figure 6:**
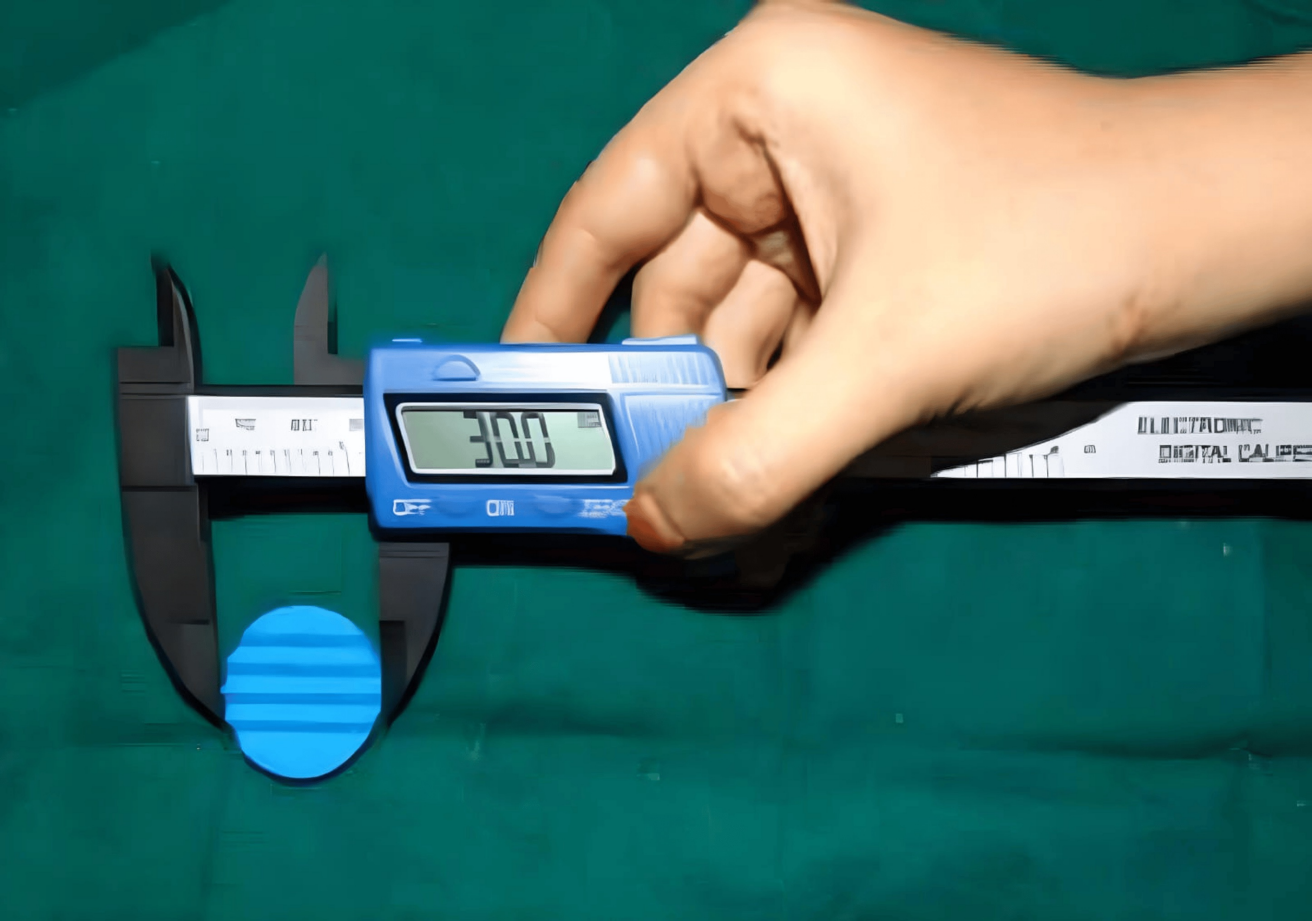
Diameter of the samples (30 mm) Image credits: Vandana Rai

**Figure 7:**
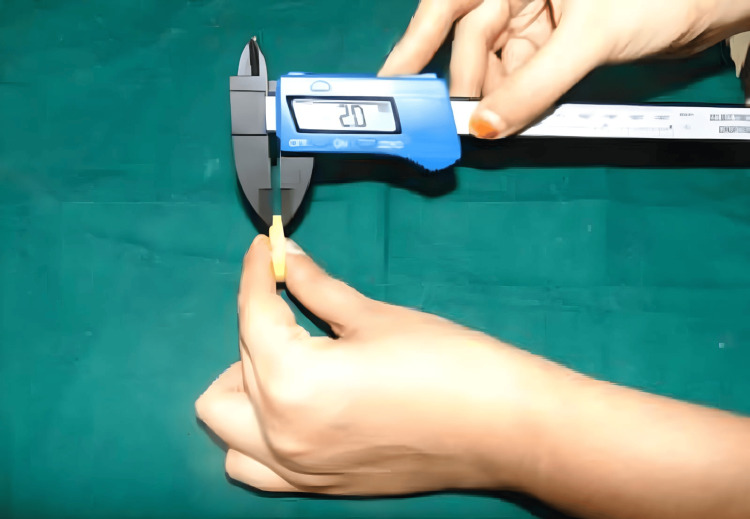
Thickness of the samples (2 mm) Image credits: Vandana Rai

Reference points A and B were marked on disk-shaped samples measuring 30 mm in diameter (Figure 6) and 2 mm in thickness (Figure 7). Using a stereomicroscope, the distance between these points was measured for 20 samples from each material group at each time interval. These values were compared to the standard distance of 20.11 mm from the stainless-steel die. The linear dimensional changes among the four interocclusal recording materials were analyzed statistically and summarized in a table.

Observation of samples for weight changes

Weight changes were measured for 20 samples of each material immediately after removal from the stainless-steel die, and again at 1 hour, 24 hours, and 1 week. An electronic scale was used to record these measurements, which were then used to calculate weight variations over time.

## Results

At intervals of 1 hour, 24 hours and 1 week, the linear dimensions and weight changes of all 80 samples were measured.

Linear dimensional changes among the materials during the interval

**Table 2:** Comparison of reference point distances in four interocclusal recording materials over time using one-way ANOVA Table credits: Kanimozhi Kulasekaran ANOVA: Analysis of variance

Time	Name of the Interocclusal Recording Material	N	Mean	SD	95% CI		F value	p value
					Lower	Upper		
1 hour	Polyether (Ramitec)	20	20.7240	0.24655	20.2236	20.4544	15.968	<0.001
	Polyvinylsiloxane (O-Bite)	20	20.7240	0.08635	20.6836	20.7644		
	Polyvinylsiloxane (Jet Bite)	20	20.2210	0.34438	20.0598	20.3822		
	Dimethacrylate (LuxaBite)	20	20.4455	0.21130	20.3466	20.5444		
24 hours	Polyether (Ramitec)	20	20.4130	0.29283	20.2760	20.5500	17.82	<0.001
	Polyvinylsiloxane (O-Bite)	20	20.7260	0.08413	20.6866	20.7654		
	Polyvinylsiloxane (Jet Bite)	20	20.2150	0.34672	20.0527	20.3773		
	Dimethacrylate (LuxaBite)	20	20.4470	0.20471	20.3512	20.5428		
1 week	Polyether (Ramitec)	20	20.4700	0.29972	20.3297	20.6103	16.11	<0.001
	Polyvinylsiloxane (O-Bite)	20	20.7250	0.08538	20.6850	20.7650		
	Polyvinylsiloxane (Jet Bite)	20	20.2100	0.34662	20.0478	20.3722		
	Dimethacrylate (LuxaBite)	20	20.4470	0.20954	20.3489	20.5451		

Interpretation

One-way ANOVA demonstrated statistically significant differences in dimensional stability among the four interocclusal recording materials at all measured time intervals 1 hour, 24 hours, and 1 week (F=15.23-17.82, p<0.001). These results indicate that the dimensional changes observed across materials are unlikely due to random variation and reflect true differences in material behavior over time. Polyvinylsiloxane materials (Jet Bite and O-Bite) consistently showed lower dimensional change, suggesting superior stability compared to polyether (Ramitec) and dimethacrylate (LuxaBite). The statistical significance across all time points reinforces the clinical importance of selecting materials with minimal dimensional drift, especially when articulation of casts is delayed beyond the initial hour.

**Table 3:** Comparison of distances between reference points at various time intervals for four interocclusal recording materials using repeated measures ANOVA Table credits: Kanimozhi Kulasekaran ANOVA: Analysis of variance

Name of the Interocclusal Recording Material	Time	No. of samples	Mean	Mean Dimensional Change in %	SD	95% CI		F value	p value
						Lower	Upper		
Polyether (Ramitec)	1 hour	20	20.7240	3.0	0.24655	20.684	20.764	2.03	0.212
	24 hours	20	20.7250	3.058	0.29283	20.687	20.765		
	1 week	20	20.7245	3.055	0.29972	20.685	20.765		
Polyvinylsiloxane (O-Bite)	1 hour	20	20.3390	1.1	0.08635	20.224	20.454	14.91	0.0006
	24 hours	20	20.4130	1.5	0.08413	20.276	20.550		
	1 week	20	20.4128	1.7	0.08538	20.330	20.610		
Polyvinylsiloxane (Jet Bite)	1 hour	20	20.2100	0.49	0.34438	20.060	20.382	0.82	0.449
	24 hours	20	20.2150	0.52	0.34672	20.053	20.377		
	1 week	20	20.2110	0.50	0.34662	20.048	20.372		
Dimethacrylate (LuxaBite)	1 hour	20	20.4455	1.668	0.21130	20.347	20.544	0.67	0.522
	24 hours	20	20.4450	1.665	0.20471	20.351	20.543		
	1 week	20	20.4450	1.665	0.20471	20.351	20.543		

Interpretation

One polyvinylsiloxane material (O-Bite) showed a statistically significant p-value (<0.05), indicating true dimensional change over time. The other (Jet Bite) was the most dimensionally stable, with a high p-value, meaning no significant variation. Polyether (Ramitec) and dimethacrylate-based composite (LuxaBite) did not show significant statistical variation but had notable clinical considerations, especially at early time points.

All four interocclusal recording materials exhibited minimal dimensional changes after one hour, though their stability differed over extended periods. Among them, one polyvinylsiloxane (Jet Bite) demonstrated the highest dimensional stability, with negligible changes recorded across all time intervals (0.49% at 1 hour, 0.52% at 24 hours, and 0.50% at 1 week). The other polyvinylsiloxane (O-Bite) showed comparable performance but with slightly greater dimensional changes (1.13%, 1.51%, and 1.50%, respectively). Dimethacrylate-based composite (LuxaBite) initially expanded (1.668% at 1 hour), likely due to polymerization-induced mass loss, and then stabilized over time. In contrast, polyether (Ramitec) exhibited the highest initial deviation (3.00% at 1 hour), followed by minimal variation thereafter.

A Friedman test with post-hoc analysis assessed time-dependent changes in dimensional stability. One polyvinylsiloxane (Jet Bite) showed no statistically significant differences across time points (p=0.449), confirming its superior stability. The other polyvinylsiloxane (O-Bite) exhibited a significant change (p=0.0006), primarily between 1 hour and 24 hours, after which it stabilized. Dimethacrylate-based composite (LuxaBite) (p=0.522) and polyether (Ramitec) (p=0.212) showed no significant differences over time, though polyether (Ramitec) exhibited relatively higher early deviations.

Overall, most materials attained dimensional stability within 24 hours, emphasizing the importance of selecting interocclusal materials based on both short-term and long-term dimensional performance. Polyvinylsiloxane materials consistently showed the least linear dimensional change across all time intervals, while polyether (Ramitec) experienced the greatest dimensional variation (Table 3).

Weight changes among the materials during the intervals

Each sample's weight was noted as soon as it was taken out of the die after 1 hour, after 24 hours, and after 1 week. 

**Table 4:** Weight comparison of samples in interocclusal recording materials at different time intervals using repeated measures ANOVA test Table credits: Kanimozhi Kulasekaran ANOVA: Analysis of variance

Name of the Interocclusal Recording Material	Time	No.of samples	Mean	SD	95% CI		F value	p value
					Lower	Upper		
Polyether (Ramitec)	0 hours	20	3.3406	0.28840	3.206	3.476	0.001	0.999
	1 hour	20	3.3406	0.28840	3.206	3.476		
	24 hours	20	3.3409	0.28897	3.206	3.476		
	1 week	20	3.3407	0.28915	3.205	3.476		
Polyvinylsiloxane (O-Bite)	0 hours	20	4.3661	0.94116	3.926	4.807	0.000	1.000
	1 hour	20	4.3661	0.94116	3.926	4.807		
	24 hours	20	4.3662	0.94125	3.926	4.807		
	1 week	20	4.3661	0.94125	3.926	4.807		
Polyvinylsiloxane (Jet Bite)	0 hours	20	4.3661	0.94116	3.926	4.807	0.000	1.000
	1 hour	20	4.3661	0.94116	3.926	4.807		
	24 hours	20	4.3663	0.99133	3.899	4.827		
	1 week	20	4.3663	0.99125	3.899	4.827		
Dimethacrylate (LuxaBite)	0 hours	20	3.3979	0.43125	3.196	3.600	0.762	0.475
	1 hour	20	3.3979	0.43125	3.196	3.600		
	24 hours	20	3.3722	0.43738	3.168	3.577		
	1 week	20	3.3720	0.43738	3.167	3.577		

Interpretation

After 1 hour, the samples in each group showed no discernible weight changes (Table 4). However, at 24 hours, different patterns emerged: Polyether (Ramitec) and polyvinylsiloxane materials (O-Bite, and Jet Bite) exhibited weight gain, while dimethacrylate (Luxabite) displayed weight loss. Among these, polyether (Ramitec) experienced the highest weight gain, followed by polyvinylsiloxane materials (Jet Bite and O-Bite). By one week, the trend shifted: Polyether (Ramitec), a polyvinylsiloxane material (O-Bite), and dimethacrylate (Luxabite) all showed weight loss, whereas the other polyvinylsiloxane material (Jet Bite) maintained a stable weight with no change observed.

Tukey’s HSD

Where statistically significant differences were identified through ANOVA, Tukey’s honestly significant difference (HSD) post-hoc test was applied to determine which specific pairs of interocclusal recording materials differed significantly. The detailed results are presented below (Tables 5-7). 

**Table 5:** Tukey’s HSD post-hoc test for dimensional change at 1 hour Table credits: Kanimozhi Kulasekaran HSD: Honestly significant difference

Material 1	Material 2	Mean Difference	p-value	95% CI Lower	95% CI Upper	Significant
Polyvinylsiloxane (Jet Bite)	Polyvinylsiloxane (O-Bite)	0.4892	<0.001	0.3043	0.6741	Yes
Polyvinylsiloxane (Jet Bite)	Polyether (Ramitec)	0.4700	<0.001	0.2851	0.6548	Yes
Polyvinylsiloxane (Jet Bite)	Dimethacrylate (LuxaBite)	0.2271	0.0098	0.0422	0.4119	Yes
Dimethacrylate (LuxaBite)	Polyvinylsiloxane (O-Bite)	0.2622	0.0021	0.0773	0.4470	Yes
Dimethacrylate (LuxaBite)	Polyether (Ramitec)	0.2429	0.0050	0.0580	0.4278	Yes
Polyvinylsiloxane (O-Bite)	Polyether (Ramitec)	-0.0193	0.9632	-0.2042	0.1656	No

**Table 6:** Tukey’s HSD post-hoc test for dimensional change at 24 hours Table credits: Kanimozhi Kulasekaran HSD: Honestly significant difference

Material 1	Material 2	Mean Difference	p-value	95% CI Lower	95% CI Upper	Significant
Polyvinylsiloxane (Jet Bite)	Polyvinylsiloxane (O-Bite)	0.3503	0.0006	0.1542	0.5464	Yes
Polyvinylsiloxane (Jet Bite)	Polyether (Ramitec)	0.2154	0.0672	-0.0111	0.4420	No
Polyvinylsiloxane (Jet Bite)	Dimethacrylate (LuxaBite)	0.0806	0.7850	-0.1459	0.3072	No
Dimethacrylate (LuxaBite)	Polyvinylsiloxane (O-Bite)	0.2696	0.0128	0.0735	0.4657	Yes
Dimethacrylate (LuxaBite)	Polyether (Ramitec)	0.1348	0.4032	-0.0613	0.3309	No
Polyvinylsiloxane (O-Bite)	Polyether (Ramitec)	-0.1348	0.4032	-0.3309	0.0613	No

**Table 7:** Tukey’s HSD post-hoc test for dimensional change at 1 week Table credits: Kanimozhi Kulasekaran HSD: Honestly significant difference

Material 1	Material 2	Mean Difference	p-value	95% CI Lower	95% CI Upper	Significant
Polyvinylsiloxane (Jet Bite)	Polyvinylsiloxane (O-Bite)	0.5134	<0.001	0.3365	0.6903	Yes
Polyvinylsiloxane (Jet Bite)	Polyether (Ramitec)	0.2593	0.0028	0.0824	0.4362	Yes
Polyvinylsiloxane (Jet Bite)	Dimethacrylate (LuxaBite)	0.2370	0.0066	0.0601	0.4139	Yes
Dimethacrylate (LuxaBite)	Polyvinylsiloxane (O-Bite)	0.2764	0.0087	0.0995	0.4533	Yes
Dimethacrylate (LuxaBite)	Polyether (Ramitec)	0.0222	0.9874	-0.1547	0.1991	No
Polyvinylsiloxane (O-Bite)	Polyether (Ramitec)	-0.2542	0.0034	-0.4311	-0.0773	Yes

Interpretation (One Hour Post-Hoc Analysis)

At the one-hour time interval, a polyvinylsiloxane (Jet Bite) demonstrated statistically significant dimensional differences when compared with the other polyvinylsiloxane (O-Bite), polyether (Ramitec), and dimethacrylate (LuxaBite), indicating superior short-term dimensional stability.

Likewise, dimethacrylate (LuxaBite) was significantly more stable than both O-Bite and Ramitec. These findings suggest that both Jet Bite and LuxaBite maintain better accuracy within the first hour of setting, making them more suitable for clinical situations where immediate articulation may be delayed.

In contrast, O-Bite and Ramitec showed no significant difference from each other, indicating similar (and comparatively lower) short-term stability. This suggests that they may be less ideal for delayed mounting scenarios.

Interpretation (24 Hours Post-Hoc Analysis)

At 24 hours, one polyvinylsiloxane (Jet Bite) remained significantly more dimensionally stable than the other (O-Bite). However, no statistically significant difference was found between Jet Bite and either polyether (Ramitec) or dimethacrylate (LuxaBite), suggesting that their performance begins to align over time.

Dimethacrylate (LuxaBite) also showed significantly better dimensional stability than O-Bite, but not compared to Ramitec. This indicates that O-Bite continues to underperform, while LuxaBite and Ramitec begin to exhibit comparable behavior to Jet Bite after 24 hours.

Overall, Jet Bite still leads in performance, but the gap between materials begins to narrow by the 24-hour mark. O-Bite consistently demonstrates the least stability, making it less suitable for clinical scenarios where cast mounting is delayed.

Interpretation (One Week Post-Hoc Analysis)

At one week, one polyvinylsiloxane (Jet Bite) continued to show statistically significant differences compared to all other materials, including the other polyvinylsiloxane (O-Bite), polyether (Ramitec), and dimethacrylate (LuxaBite). This indicates that Jet Bite maintains superior dimensional stability even after prolonged periods, making it the most reliable choice when delayed articulation is required.

Dimethacrylate (LuxaBite) also showed significant differences compared to O-Bite, suggesting better long-term performance. However, its difference with Ramitec was not statistically significant, indicating similar behavior between LuxaBite and Ramitec at the one-week mark.

One polyvinylsiloxane (O-Bite) showed the greatest dimensional change over time, with significant differences from both Jet Bite and LuxaBite, and even from Ramitec. This reinforces its unsuitability for cases requiring storage or delayed cast mounting.

## Discussion

This study evaluated the dimensional and weight changes of four interocclusal recording materials, namely, polyether (Ramitec), polyvinylsiloxanes (Jet Bite and O-Bite), and dimethacrylate (LuxaBite) at intervals of 1 hour, 24 hours, and 1 week. The objective was to determine which material provides the most dimensionally stable and clinically accurate recording.

Freilich et al. highlighted that interocclusal relationships can be recorded using direct, functional, or graphic methods [4]. However, the choice of recording material is primarily influenced by its ability to provide vertical support and horizontal stability,both of which are key for accurate and repeatable cast articulation.

Although no single material fulfills all ideal requirements, the most clinically effective interocclusal recording materials are characterized by low viscosity, minimal resistance to closure, high accuracy, rapid setting times, ease of manipulation, and, most importantly, dimensional stability. Among these, dimensional stability and accuracy are particularly vital, as they directly influence the fidelity of the maxillomandibular relationship captured in the record.

Lassila’s research underscores the importance of dimensional stability in elastomeric interocclusal materials, noting that while they tend to maintain their dimensions over time, they are susceptible to significant expansion when exposed to moisture, thus, emphasizing the need for proper handling and storage to avoid dimensional distortion due to humidity [5].

Further, existing studies indicates that silicone-based impression materials can undergo shrinkage due to polymerization and the evaporation of volatile components [6,7]. This process can lead to weight loss, which is typically a result of the release of volatile substances. Conversely, weight gain in these materials often suggests sensitivity to humidity, which can compromise their dimensional accuracy.

Among the materials evaluated in this study, a polyvinylsiloxane (Jet Bite) demonstrated the highest precision, exhibiting minimal dimensional changes across all measured time intervals (0.49% at 1 hour, 0.52% at 24 hours, and 0.50% at 1 week). The slight contraction observed can likely be attributed to polymerization shrinkage and the formation of byproducts. Moreover, this material showed no significant weight fluctuations at any interval, with only a slight weight increase at 24 hours, which could be linked to moisture absorption.

On the other hand, the other polyvinylsiloxane material (O-Bite), demonstrated superior accuracy and dimensional stability compared to polyether and dimethacrylate (Ramitec and LuxaBite) but did not surpass Jet Bite's performance. This material demonstrated expansion at 1 hour, 24 hours, and 1 week, followed by a contraction phase. This pattern of expansion corresponds to the initial weight gain observed at 24 hours, which was followed by weight loss at 1 week. The weight reduction could be attributed to the release of hydrogen, a well-documented phenomenon in silicone materials. While many manufacturers incorporate palladium or platinum as scavengers in polyvinylsiloxane impression materials to counter hydrogen release, there is a lack of published research confirming the presence of scavengers in interocclusal registration materials, possibly because these materials do not require gypsum pouring [2,8].

Also, the methacrylate-based interocclusal recording material demonstrated superior accuracy compared to polyether. It exhibited an initial expansion after 1 hour, followed by contraction at 24 hours, with no further dimensional changes noted from 24 hours to 1 week. The observed expansion may be attributed to the exothermic reaction occurring during polymerization, while the subsequent contraction is likely a result of molecular densification associated with ongoing polymerization and cross-linking.This finding is consistent with the weight loss observed at both 24 hours and 1 week, which can be linked to the loss of volatile components during the curing process.

Among the materials evaluated, polyether (Ramitec) exhibited the most significant deviation from the stainless-steel die. Its hydrophilic nature allows it to absorb moisture, leading to significant dimensional expansion and considerable weight changes within just 24 hours which is consistent with the findings of Muller et al. and Braden et al. where this material exhibited increase in dimensional changes after 6 to 24 hours of storage, which is likely attributable to the expulsion of water following the polymerization process [8,9].

Overall, this study supports the findings of Breeding et al., which demonstrated that incorporation of polyvinylsilicones significantly reduces mounting errors during interocclusal recordings compared to acrylic and thermoplastic resins [10]. Additionally, our results are consistent with the research conducted by Dua et al., indicating that polyether materials experience greater dimensional changes relative to addition silicone interocclusal materials [11]. In a related investigation, Chandu et al. who evaluated the compressive resistance of interocclusal recording materials, identified polyvinylsiloxane as exhibiting the highest compressive resistance, thereby providing enhanced stability under pressure compared to polyether materials [12].

However, some studies, such as those conducted by Gurav et al. and Patel et al., have reported contrasting findings regarding dimensional stability [7,13]. Gurav et al. noted no significant difference between polyether and polyvinylsiloxane materials, while Patel et al. found that among five interocclusal materials, including methacrylate-based, polyvinylsiloxane, and polyether materials, LuxaBite (methacrylate) exhibited the least dimensional change and the highest stability following immersion in disinfectants [7,13]. These discrepancies highlight the complex interactions between material composition and environmental factors affecting dimensional stability. Despite these conflicting results, the overall evidence supports the conclusion that polyvinylsiloxane materials generally provide enhanced performance in clinical settings, offering greater reliability for achieving precise restorations.

In conclusion, this study indicates that polyvinylsiloxane interocclusal recording materials (JetBite and O-Bite) exhibit the lowest margin of error. They facilitate easy manipulation without the need for a carrier in the mouth, demonstrate minimal resistance during closure, set to a trimmable consistency, and accurately reproduce intricate dental details. Given the significant dimensional changes observed within the first hour following interocclusal registration, it is advisable that polyvinylsiloxane materials should be articulated within 24 hours, while polyether should be articulated immediately. Additionally, dimethacrylate material also show dimensional changes within the first hour, albeit to a lesser extent than polyether, emphasizing the importance of early articulation to ensure precise restorations and minimize distortion.

Limitations

This in vitro study was conducted under standardized laboratory conditions and did not replicate certain intraoral factors such as saliva, temperature fluctuations, and masticatory forces, which may influence material behavior in clinical settings. The evaluation was limited to linear dimensional and weight changes; other clinically relevant parameters-such as elastic recovery, three-dimensional distortion, setting time, compressive strength, and handling characteristics-were not assessed. Additionally, the absence of examiner blinding and inter-examiner calibration could introduce potential measurement variability. These limitations should be considered when interpreting the results, and future studies incorporating clinically simulated environments and broader material performance criteria are encouraged.

Recommendations

Articulation of polyvinylsiloxane materials should be done within 24 hours, whereas for polyether and dimethacrylate materials articulation should be done immediately.

Clinical implications

One polyvinylsiloxane (Jet Bite) showed superior dimensional stability, making it ideal when delayed articulation is needed. In contrast, polyether (Ramitec) and dimethacrylate (LuxaBite) showed more dimensional changes in the first hour and are recommended only for immediate articulation. This highlights the importance of selecting materials based on clinical conditions and timing of cast mounting to minimize occlusal errors.

## Conclusions

Within the limitations of this in vitro study, and among the materials tested, polyvinylsiloxane-based materials (Jet Bite and O-Bite) demonstrated the highest dimensional and weight stability over time. These materials may be considered suitable for clinical situations where articulation is delayed up to 24 hours. In contrast, polyether (Ramitec) and dimethacrylate-based composite (LuxaBite) exhibited more pronounced changes within the first hour, suggesting that immediate articulation is recommended to minimize potential inaccuracies. These findings support the importance of selecting interocclusal materials based on their time-dependent stability characteristics.

## References

[1] Michalakis KX, Pissiotis A, Anastasiadou V, Kapari D (2004). An experimental study on particular physical properties of several interocclusal recording media. Part II: linear dimensional change and accompanying weight change. J Prosthodont.

[2] Michalakis KX, Pissiotis A, Anastasiadou V, Kapari D (2004). An experimental study on particular physical properties of several interocclusal recording media. Part I: consistency prior to setting. J Prosthodont.

[3] Malone WF, Koth DL, Kaiser DA, Cavazos Cavazos, Morgano SM (1997). Tylman’s Theory and Practice of Fixed Prosthodontics, 8th ed.. https://www.amazon.in/Tylmans-Theory-Practice-Fixed-Prosthodontics/dp/8185502145.

[4] Freilich MA, Altieri JV, Wahle JJ (1992). Principles for selecting interocclusal records for articulation of dentate and partially dentate casts. J Prosthet Dent.

[5] Lassila V (1986). Comparison of five interocclusal recording materials. J Prosthet Dent.

[6] Lepe X, Johnson GH, Berg JC, Aw TC, Stroh GS (2002). Wettability, imbibition, and mass change of disinfected low-viscosity impression materials. J Prosthet Dent.

[7] Gurav SV, Khanna TS, Nandeeshwar DB (2015). Comparison of the accuracy and dimensional stability of interocclusal recording materials: an in vitro study. Int J Sci Res Publ.

[8] Müller J, Götz G, Hörz W, Kraft E (1990). Study of the accuracy of different recording materials. J Prosthet Dent.

[9] Braden M, Causton B, Clarke RL (1972). A polyether impression rubber. J Dent Res.

[10] Breeding LC, Dixon DL, Kinderknecht KE (1994). Accuracy of three interocclusal recording materials used to mount a working cast. J Prosthet Dent.

[11] Dua P, Gupta SH, Ramachandran S, Sandhu HS (2007). Evaluation of four elastomeric interocclusal recording materials. Med J Armed Forces India.

[12] Chandu GS, Khan MF, Mishra SK, Asnani P (2015). Evaluation and comparison of resistance to compression of various interocclusal recording media: an in vitro study. J Int Oral Health.

[13] Patel RK, Pai SA, Nagaraj T, Kohli A, Mangala Jyothi KJ, Smitha BG (2019). Linear dimensional changes of five interocclusal recording materials when immersed in two disinfectants for different time intervals. J Contemp Dent Pract.

